# A Case Report on Parvovirus B19 Associated Myositis

**DOI:** 10.1155/2012/250537

**Published:** 2012-12-10

**Authors:** Nathan D. Oliver, Auleen Millar, Adrian Pendleton

**Affiliations:** Department of Rheumatology, Musgrave Park Hospital, Belfast Health and Social Care Trust, Queen's University Belfast, Nathan Oliver, Killuney House, Portadown Road, Armagh BT61 9HE, UK

## Abstract

*Introduction*. Whilst there are reports of viral myopathies affecting children and the immunocompromised, infective myositis is a relatively rare inflammatory myopathy in adults. The clinical spectrum can range from benign myalgias to more serious complications in certain risk groups. *Case Presentation*. We present two cases of myositis as a result of parvovirus B19 infection. *Conclusion*. Viral myositis and parvovirus B19 associated myositis should be considered in adults presenting with significant myalgia.

## 1. Introduction

Myositis is an inflammatory myopathy mainly associated with viral infection. The spectrum formed by the disease means presentation can range from vague muscular pain, swelling, and oedema to cardiac arrhythmias as a result of electrolyte disturbances from severe rhabdomyolysis.

Of the suspected viral causes of myositis, parvovirus B19 is one of the least frequently recorded. This is thought to be a result of the acquired immunity developed by most adults over their lifetime. It follows that parvovirus B19 infects children more frequently; however, children rarely develop anything more than erythema infectiosum before recovery [1]. We describe two adult cases that developed myositis as a result of parvovirus B19 infection.

## 2. Case Presentation

The first patient is a 42-year-old Caucasian male who presented with a four-week history of pain in his calves associated with inflammation, this soon progressed to involve his thighs and had an associated weakness. He further described ankle swelling, bilateral forearm pain, nocturnal hyperhidrosis, and recent contact with children diagnosed with “slapped cheek syndrome” associated with erythrovirus B19 infection.

Physical examination revealed bilateral calf muscle hypertrophy, with pitting oedema of the ankles. Both lower limbs had decreased power (4/5) in all muscle groups. Initial blood tests revealed an elevated C-reactive protein and normal creatinine kinase. A muscle biopsy of the left thigh was performed yielding no pathological diagnosis, and a T2 and short TI inversion recovery MRI revealed bilateral marked intramuscular septal oedema of the soleus muscles and subcutaneous oedema of the lower legs. EMG showed mild myopathic changes but no spontaneous fibrillation. HIV/Hep B/C tests were negative; however, muscle biopsy was seen to be PCR positive for erythrovirus B19 with 32900 cop/m. The patient commenced prednisolone with significant clinical response within 2 weeks.

The second patient is a 46-year-old Caucasian male with a background of antiphospholipid syndrome who presented with a six-week history of sudden onset of bilateral elbow pain which progressed to involve both calves. Inflammatory markers were raised with erythrocyte sedimentation rate 119 mm/hr, C-reactive protein 34 mg/L, and creatinine kinase 187 IU/L.

**Figure 1:**
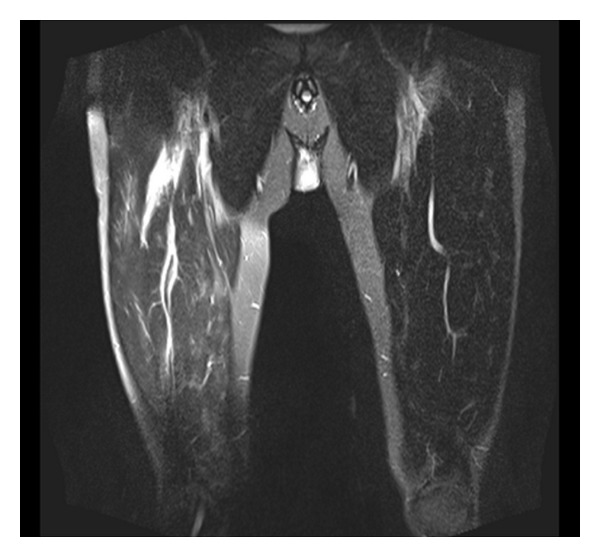
Coronal STIR MRI image showing high signal change in the right thigh associated with intramuscular oedema.

On examination the only positive finding was tenderness over the anterior quadriceps and calf muscles. MRI showed significant intramuscular oedema in both legs (Figure 1). Erythrovirus B19 IgM was positive, and PCR quantification indicated strong positivity at 66400 cop/m. The patient was started on 40 mg prednisolone PO OD, and on review one week later the patient was significantly improved.

## 3. Discussion

Parvovirus B19 as a cause of myositis is rare and seldom reported. Indeed, the first report was in rheumatology in 2000 [2]. In this case the authors described a patient with inflammatory myopathy and parvovirus B19 DNA in two muscle biopsies five months apart. Since then, there have been as many reports denying any association between parvovirus B19 and myositis, as there have been to support it. Indeed, some of the authors involved in the aforementioned paper in support of an association have now come to believe there is no link between myositis and parvovirus B19 [3]. This is because initial findings of the presence of parvovirus B19 in the musculature of one patient with dermatomyositis have been superseded by the lack of identification of B19 DNA in muscle biopsies in any of 8 patients with inflammatory myopathies.

In support of the association, the first case report is strengthened by the fact that no other identifiable cause for the dermatomyositis other than parvovirus B19 was found, and the patient recovered with prednisolone and methotrexate treatment. However, the second case report undermines this slightly as it shows a lack of any B19 in any of 8 patients known to have inflammatory myopathy.

It has been shown that some 15% of newly diagnosed arthritis is most likely caused by parvovirus infection [4]. We now present two cases of acute inflammatory myositis without arthritis in association with acute erythrovirus B19 infection. We thus propose that there is a true association with inflammatory myositis.

We highlight these cases to support the debate as to a true association between parvovirus B19 infection and myositis, but agree that more scientific study into the field is needed.

## 4. Conclusion

Parvovirus B19 infection is rare in adults. However, it should be considered a potential causative factor in patients with myositis. A larger-scale epidemiological study would be useful to help ascertain whether, in fact, there is a true association between parvovirus B19 infection and myositis.
